# Supplementary material for: Ultrasound-Responsive Liposomes for Targeted Drug Delivery Combined with Focused Ultrasound
Source: Pharmaceutics. 2022 Jun 21;14(7):1314. doi: 10.3390/pharmaceutics14071314 (PMC9315635; doi:10.3390/pharmaceutics14071314)

<Supporting information>

Ultrasound-responsive liposomes for targeted drug delivery combined with focused ultrasound

Yoon-Seok Kim, Min Jung Ko, Hyungwon Moon, Wonchul Sim, Ae shin Cho, Gio Gil and Hyun Ryoung Kim *

R&D center, IMGT Co., Ltd, 172, Dolma-ro, Bundang-gu, Seongnam-si, Gyeonggi-do, Republic of Korea

* Correspondence: hyunryoung.kim@nanoimgt.com

Keywords: ultrasound-responsive liposome; liposomal doxorubicin; stimuli-triggered drug release; high-intensity focused ultrasound; triple negative breast cancer

Figure S1. (a) Doxorubicin release ratio of IMP301 dependent on the temperature, (b) simulation of temperature increases dependent on the focused ultrasound condition.

(a)

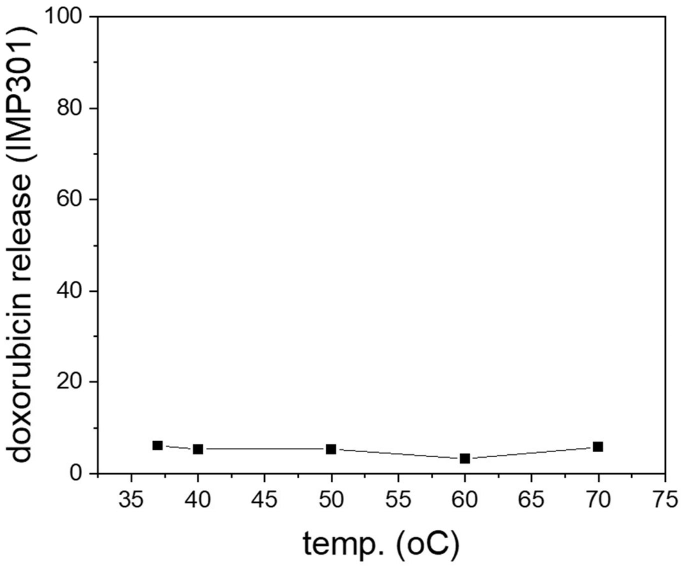

(b)

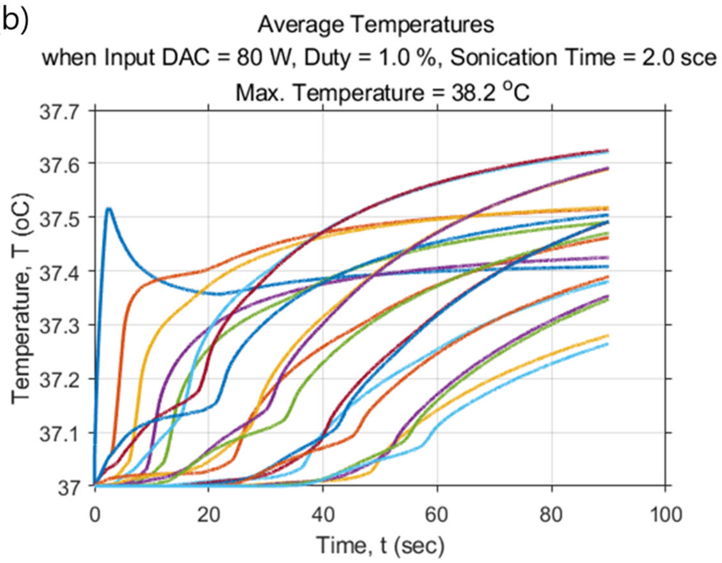

Time(sec)		2	4	6	8	10	12	14	16	18	20	22	24	26	28	30
Duty	1%	37.6	38.0	38.2	38.4	38.6	38.7	38.8	38.9	39.0	39.1	39.1	39.2	39.2	39.3	39.3
	2%	38.1	38.7	39.1	39.5	39.8	40.0	40.2	40.4	40.6	40.7	40.9	41.0	41.1	41.2	41.3
Time(sec)		32	34	36	38	40	42	44	46	48	50	52	54	56	58	60
Duty	1%	39.4	39.4	39.5	39.5	39.5	39.6	39.6	39.6	39.7	39.7	39.7	39.7	39.8	39.8	39.8
	2%	41.4	41.5	41.6	41.6	41.7	41.8	41.8	41.9	42.0	42.0	42.1	42.1	42.2	42.2	42.2

Figure S2. Population of free DOX to heart at 2h of post administration of DOX, DOXIL and IMP301.

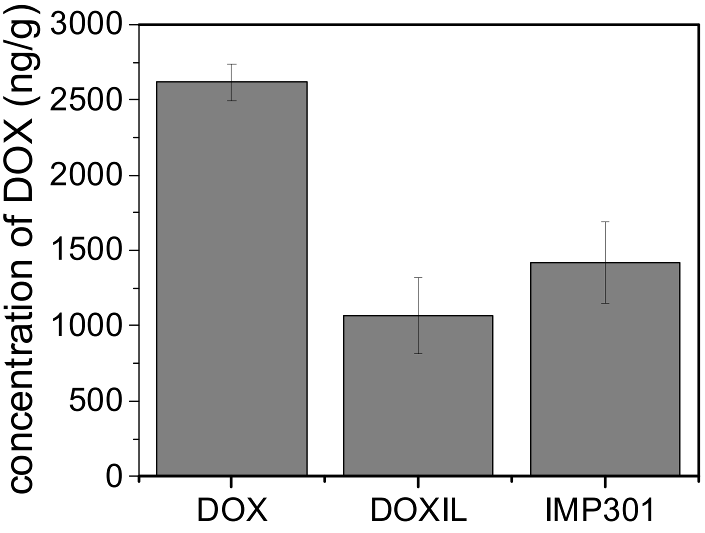

Supplement: Supplementary file 1 [file pharmaceutics-14-01314-s001.zip › pharmaceutics-1755191-supplementary.pdf]
